# Prediction of bile salt export pump inhibition using biomimetic chromatographic descriptors: Integrating membrane affinity and protein binding

**DOI:** 10.5599/admet.3303

**Published:** 2026-05-24

**Authors:** Chrysanthos Stergiopoulos, Klara Valko

**Affiliations:** 1 Laboratory of Process Analysis and Design, School of Chemical Engineering, National Technical University of Athens, Iroon Polytechniou 9, 157 75 Zografou, Athens, Greece; 2 Bio-Mimetic Chromatography Ltd, Business & Technology Centre, Bessemer Drive, Stevenage, Herts, SG1 2DX, United Kingdom

**Keywords:** Hepatotoxicity, canalicular transport, predictive modelling, drug distribution, transporter inhibition

## Abstract

**Background and purpose:**

Bile salt export pump (BSEP) inhibition is a key mechanistic driver of cholestatic drug-induced liver injury, yet current prediction approaches rely mainly on in vitro potency and calculated physicochemical descriptors that do not adequately reflect local exposure at the canalicular membrane. This study aimed to evaluate whether biomimetic chromatographic descriptors could improve predictions of BSEP inhibition by incorporating mechanistically relevant information on membrane affinity and protein binding.

**Experimental approach:**

A dataset of 62 structurally diverse compounds with reported BSEP inhibition data was compiled and modelled using multiple linear regression. Biomimetic descriptors derived from immobilized artificial membrane chromatography and human serum albumin affinity (log *k*_HSA_) were compared against conventional descriptors such as lipophilicity and molecular weight. Model performance was assessed using internal and external validation, graphical diagnostics, Y-randomization, and applicability domain analysis.

**Key results:**

Models based on biomimetic descriptors demonstrated superior, more balanced predictive performance than conventional physicochemical models, with greater robustness and external predictivity. The combination of membrane-affinity and protein-binding descriptors provided a more accurate representation of the determinants governing BSEP interactions.

**Conclusion:**

Biomimetic chromatographic descriptors provide an experimentally grounded approximation of membrane-proximal exposure, a key determinant of BSEP interactions that conventional descriptors do not capture. This approach extends beyond traditional quantitative structure-activity relationship by incorporating distribution-relevant properties into predictive modelling and offers a simple, interpretable framework to support early identification of transporter-mediated toxicity risk in drug discovery.

## Introduction

Drug-induced liver injury (DILI) remains a leading cause of drug attrition and post-marketing withdrawal, with cholestatic and mixed phenotypes frequently linked to disruption of bile acid homeostasis [1]. The bile salt export pump (BSEP; ABCB11), an ATP-dependent transporter located at the canalicular membrane of hepatocytes, represents the rate-limiting step in bile acid secretion. Inhibition of BSEP can result in intracellular accumulation of bile acids, triggering hepatocellular stress, inflammation, and ultimately cholestasis [2]. Clinical and genetic evidence, including progressive familial intrahepatic cholestasis type 2 (PFIC2), supports a causal role of impaired BSEP function in liver injury [1,2].

In pharmaceutical development, BSEP inhibition is therefore considered a critical mechanistic liability [1,2]. However, translation of in vitro BSEP inhibition data into clinical risk remains challenging [1,3]. Standard vesicle-based assays provide estimates of inhibitory potency (*e.g.* IC_50_), but their predictive value is limited by several factors, including uncertainty in unbound concentrations, the absence of metabolic processes, and limited representation of hepatocellular drug accumulation [1,4]. Importantly, clinical DILI risk is not governed solely by inhibitory potency, but by the interplay between potency and effective exposure at the site of interaction [1,3,4]. As a result, contemporary frameworks emphasize exposure-adjusted metrics (*e.g.* Css/IC_50_: steady-state systemic exposure divided by the *in vitro* inhibitory concentration) yet estimating relevant intracellular and membrane-proximal concentrations remains a major unresolved challenge [1,2,4]. Notably, BSEP inhibition alone is not a universal predictor of clinical DILI, as hepatotoxic risk is modulated by additional factors, including intracellular accumulation, metabolism, and mitochondrial liability [3].

Several computational models have been developed to predict BSEP inhibition, including quantitative structure-activity relationship (QSAR) models, machine learning approaches, and structure-based methods. QSAR and classification models based on molecular descriptors and physicochemical properties have demonstrated moderate-to-good predictive performance for BSEP inhibition [5-12]. More recently, machine learning and deep learning approaches, including support vector machines and graph-based neural networks, have further improved prediction accuracy by capturing nonlinear structure-activity relationships [9,12]. Their broader predictive utility has historically been constrained by limited structural information, although recent structural studies have begun to clarify the molecular basis of bile salt extrusion and small-molecule inhibition in human BSEP [13].

Despite these advances, existing models primarily rely on calculated molecular descriptors or structural features and often do not explicitly incorporate experimentally derived distribution parameters such as membrane partitioning and plasma protein binding [4,14]. This is important because BSEP inhibition depends not only on intrinsic inhibitory potency, but also on the effective local concentration of the compound at the canalicular membrane and the unbound fraction available for transporter interaction [1,2,4]. Conventional physicochemical descriptors, such as log *P* and log *D*, provide useful approximations of lipophilicity and distribution behaviour, but they do not fully capture microenvironmental processes such as phospholipid affinity, protein binding, and ionization-dependent interactions [4,14]. Consequently, experimentally derived descriptors that reflect these properties may improve both the mechanistic interpretation and prediction of BSEP inhibition [1,4,14].

Biomimetic chromatography provides a practical and experimentally grounded approach to address this gap [14]. Immobilized artificial membrane (IAM) chromatography enables the quantification of phospholipid affinity, serving as a proxy for membrane partitioning and potential enrichment near membrane-bound targets such as BSEP [14,15,17]. Complementarily, human serum albumin (HSA) affinity chromatography provides insight into plasma protein binding, a key determinant of unbound systemic exposure and hepatic drug availability [14,15]. Together, these two orthogonal descriptors capture fundamental aspects of drug distribution: membrane affinity (IAM) and free fraction modulation (HSA) [14-16].

Although biomimetic chromatography has been successfully applied in pharmacokinetic modelling, including the prediction of volume of distribution and tissue binding, its application to transporter-mediated toxicity, particularly BSEP inhibition, remains comparatively underexplored relative to QSAR and machine learning approaches [14,16]. Given that effective inhibition depends on both intrinsic potency and local exposure at the membrane interface, experimentally derived IAM and HSA retention parameters may provide mechanistically relevant predictors that complement traditional in vitro assays [1,4,14].

While the ultimate concern in drug discovery is clinical drug-induced liver injury (DILI), the present study focuses on BSEP inhibition as a mechanistically defined and experimentally tractable endpoint [1-3]. BSEP inhibition is a well-established mechanistic contributor to cholestatic DILI and is supported by clinical, genetic, and regulatory evidence as a key liability in drug development [1,2]. In contrast, clinical DILI is a multifactorial and heterogeneous outcome influenced by numerous factors, including metabolism, immune response, mitochondrial toxicity, exposure, dose, and patient-specific variability, thereby complicating direct modelling and reducing mechanistic interpretability [1,3,4]. Consequently, mechanistic intermediate endpoints such as BSEP inhibition provide a more controlled and interpretable framework for investigating structure-activity and distribution-toxicity relationships [1,4]. Direct modelling of clinical DILI would be valuable as a later-stage objective but would require larger curated datasets and additional descriptors that reflect complementary toxicity mechanisms [3,4]. The present approach is therefore positioned as a mechanistically informed screening layer that complements, rather than replaces, integrated DILI risk assessment strategies [1,3,4].

In this study, we propose an experimentally grounded, chromatography-informed modelling framework to predict BSEP inhibition using IAM and HSA descriptors. By integrating experimentally measured phospholipid and protein affinity with statistical modelling approaches, we aim to (i) improve prediction of BSEP inhibitory potency, (ii) capture microenvironmental exposure effects not represented by conventional physicochemical parameters, and (iii) provide an interpretable and scalable tool for early compound prioritization. This approach is positioned as a decision-support layer within an integrated DILI risk assessment, supporting a more informed selection of compounds for confirmatory transporter- and hepatocyte-based assays.

## Experimental

### Dataset and endpoint definition

A dataset comprising 62 structurally diverse compounds with reported BSEP inhibition data (IC_50_ values) was compiled from published in vitro vesicle-based transporter assays and curated datasets, primarily from Morgan *et al.* [6], Pedersen *et al.* [7], Montanari *et al.* [8], and Dawson *et al.* [18]. When multiple values were available, preference was given to data obtained under comparable experimental conditions, and inconsistent entries were excluded to ensure dataset reliability. The endpoint was expressed as pBSEP, defined as the negative logarithm of the experimental BSEP inhibition metric (IC_50_), standardized to a consistent molar concentration unit (M). Prior to analysis, chemical structures were curated to ensure consistency in representation.

The dataset was divided into a training set (*n* = 50) and an external test set (*n* = 12) using response-based stratified sampling. Specifically, compounds were first sorted by their pBSEP values, and samples were then randomly assigned to the two subsets to ensure that both subsets adequately covered the full range of the response variable. Summary statistics confirmed comparable distributions between the training and test sets in terms of range, mean and variability. The training set was used for model development and internal validation, while the test set was used exclusively for external validation. Literature pBSEP data for each set are included in Table S1 in the Supplementary material.

### Biomimetic chromatographic descriptors

Biomimetic chromatographic descriptors related to phospholipid affinity and plasma protein binding were determined using established HPLC-based methodologies described in the literature [14-16]. These descriptors were selected for their mechanistic relevance to drug-membrane interactions and protein binding, which are key factors influencing BSEP inhibition. The derived chromatographic descriptors, CHI IAM and log *k*_HSA_, were used as proxies for membrane partitioning and plasma protein binding, respectively, in subsequent QSAR modelling of BSEP inhibition. All compounds were obtained from commercial sources (Sigma-Aldrich, Merck) and prepared as 10 mM stock solutions in dimethyl sulfoxide (DMSO). Prior to analysis, samples were appropriately diluted and injected into an Agilent 1100 HPLC system equipped with a diode array detector (DAD) (Agilent Technologies, Santa Clara, CA, USA).

### Phospholipid binding (immobilized artificial membrane chromatography)

Phospholipid affinity was evaluated using an immobilized artificial membrane (IAM) column (IAM.PC.DD2, 100×4.6 mm), which mimics the phospholipid environment of biological membranes [14,16]. Chromatographic measurements were performed using a 50 mM ammonium acetate buffer (pH 7.4) as the aqueous phase and acetonitrile as the organic modifier. A gradient was applied to reach 90 % acetonitrile within 4.75 min, followed by a short hold and re-equilibration, resulting in a total run time of 6 min. Retention times were calibrated using a set of reference compounds with established CHI IAM values reported in the literature. The calibration exhibited excellent linearity between gradient retention times and CHI IAM values (*R*^2^ > 0.99), confirming the method's robustness. Measurement reproducibility was within ±0.005 min. The Chromatographic Hydrophobicity Index for IAM (CHI IAM) approximates the effective organic modifier concentration at elution and serves as a descriptor of phospholipid binding [14,16]. CHI IAM descriptors for this study’s dataset are included in Table S2 in the Supplementary material.

### Plasma protein binding (human serum albumin chromatography)

Plasma protein binding was assessed using a Chiralpak HSA column (50×3 mm, 5 μm). The mobile phase consisted of 50 mM ammonium acetate buffer (pH 7.4), with isopropanol as the organic modifier, and the gradient reached 35 % within 3.5 min [14,15]. The total analysis time was 6 min, including re-equilibration. Retention times were calibrated using reference compounds with known plasma protein-binding values reported in the literature. The calibration demonstrated strong linear correlation (*R*^2^ > 0.99), supporting the reliability of the derived descriptors. The reproducibility of retention time measurements was within ±0.01 min. The chromatographic retention data were converted to logarithmic retention factors (log *k*_HSA_), which provide an estimate of a compound's affinity for human serum albumin [15]. Chromatographic HSA affinity descriptors are summarized in Table S3 in the Supplementary material.

### In silico descriptors

Calculated physicochemical descriptors were obtained using the widely used and validated ACD/Percepta Platform (Advanced Chemistry Development, Toronto, Canada) for predicting physicochemical properties. The selected descriptors included lipophilicity (log *P*), distribution coefficient at physiological pH (log *D*_7.4_), molecular weight (MW), hydrogen-bond acceptors (HBA), hydrogen-bond donors (HBD), ionization state (net charge at pH 7.4), Abraham solvation parameters (A and B), and topological polar surface area (TPSA). These descriptors were selected for their established relevance and statistical significance in quantitative structure-activity/property/toxicity relationship (QSAR/QSPR/QTAR) studies, particularly in modeling drug distribution, membrane interactions, and transporter-related effects. The conventional lipophilicity descriptors log *P* and log *D*_7.4_ were included as reference physicochemical parameters for comparison with the experimentally derived biomimetic descriptors. Log *P* describes the partitioning behavior of the neutral form of a compound and therefore does not account for ionization at physiological pH. Log *D*_7.4_ partially addresses this limitation by representing the apparent distribution of all ionization states at pH 7.4. However, both descriptors remain bulk physicochemical parameters and do not explicitly capture microenvironmental interactions such as phospholipid affinity or plasma protein binding. All calculations were performed using standardized molecular structures under default software settings. The complete set of calculated descriptors for all compounds is provided in Table S4 in the Supplementary material.

### Model development

Quantitative structure-activity relationship (QSAR) models were developed using multiple linear regression (MLR) implemented in IBM SPSS Statistics v27 (IBM Corp., Armonk, NY, USA). Model development was restricted to the training set. Several descriptor combinations were explored, including biomimetic descriptors (CHI IAM, log *k*_HSA_), hybrid models incorporating physicochemical data, and baseline models based on conventional lipophilicity descriptors (log *P*, log *D*_7.4_). Model selection was based primarily on predictive performance, particularly external validation, rather than solely on goodness-of-fit, in accordance with established QSAR modelling and validation principles [19]. The general pattern of the model is presented by Equation (1):



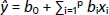

(1)


where *ŷ* is the predicted pBSEP, *b*₀ is the intercept, *b*ᵢ are the regression coefficients and *x*ᵢ are the selected descriptors.

### Cross-validation

Internal validation was performed using 5-fold cross-validation on the training set [19]. The dataset was partitioned into five subsets, and each subset was used once as validation data, with the remaining subsets used for model training. Predictions from all folds were combined to obtain cross-validated predictions.

The cross-validated predictive coefficient (*Q*^2^_cv_) was calculated by Equation (2):



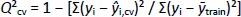

(2)


where *y*ᵢ represents the observed values, *ŷ*_i,cv_ the predicted values obtained from cross-validation, and *ȳ*_train_ the mean of the observed values in the training set.

The root-mean-square error of cross-validation (RMSECV) was calculated by Equation (3):



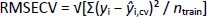

(3)


where *n*_train_ is the number of compounds in the training set.

### External validation

The final model was developed using the entire training set and applied to the external test set.

The external predictive coefficient (*Q*^2^_ext_) was calculated by Equation (4):



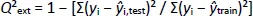

(4)


where *ŷ*ᵢ,_test_ are the predicted values for the external test set.

The root-mean-square error of prediction (RMSEP) was calculated by Equation (5):



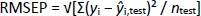

(5)


where *n*_test_ is the number of compounds in the external test set.

### Goodness-of-fit

The coefficient of determination (*R*^2^) was calculated by Equation (6):



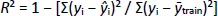

(6)


where *y*ᵢ represents the observed values, *ŷ*ᵢ the predicted values from the model, and *ȳ*_train_ the mean of the observed values in the training set.

The adjusted coefficient of determination (*R*^2^_adj_) was calculated by Equation (7):



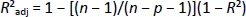

(7)


where *n* is the number of compounds in the training set, and *p* is the number of descriptors included in the model. Additional statistical parameters, including the correlation coefficient (*R*), *F*-statistic, and standard error of estimate (*s*), were evaluated to assess model robustness and statistical significance.

### Residual analysis and Y-randomization

Residual analysis was performed by plotting standardized residuals (*r*ᵢ) against predicted values. Standardized residuals, expressed in units of standard deviation, were expected to be randomly distributed around zero with no systematic patterns. A random scatter of residuals without discernible trends was considered indicative of model adequacy, linearity, and absence of heteroscedasticity.

Y-randomization (response permutation) was performed by randomly shuffling the response variable while keeping the descriptor matrix unchanged [19]. Multiple randomized models were generated and compared with the original model. A significant reduction in *R*^2^, *Q*^2^, and other predictive metrics for the randomized models confirmed that the original model was not due to chance correlation. Additionally, the intercept values of the randomized models were examined to ensure the absence of systematic bias.

### Applicability domain

The applicability domain of the final model was evaluated using the Williams plot, where standardized residuals (*r*ᵢ) were plotted against leverage values (*h*ᵢ) [19].

The warning leverage threshold (h*) was defined by Equation (8):



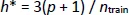

(8)


where *p* is the number of descriptors in the model and *n*_train_ is the number of compounds in the training set.

Compounds with standardized residuals |*r*ᵢ| > 3 were considered response outliers, while compounds with leverage values *h*ᵢ > h* were considered structurally influential. The absence of compounds exceeding both thresholds supported the reliability and robustness of the model predictions within its applicability domain. Compounds falling within these boundaries were within the model’s applicability domain and therefore associated with reliable predictions.

## Results and discussion

### Dataset characteristics

The dataset comprised 62 structurally diverse compounds with reported BSEP inhibition values expressed as pBSEP. While the dataset size is modest, it is consistent with experimentally derived descriptor studies, in which measurement fidelity and mechanistic interpretability are prioritized over dataset size. The response variable exhibited a broad distribution, ranging from 3.00 to 5.60, with a mean value of 4.42 and a standard deviation of 0.68, indicating substantial variability suitable for model development.

**Figure 1.**
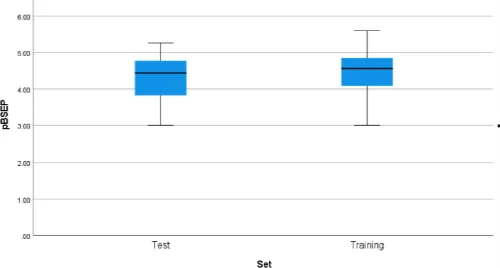
Boxplot representation of pBSEP values for the training (*n* = 50) and external test (*n* = 12) sets. The similar distributions, central tendencies, and spreads indicate a balanced dataset split and adequate coverage of the response space

The dataset was divided into a training set (*n* = 50) and an external test set (*n* = 12) using a response-based stratified sampling approach. The distribution of pBSEP values in both subsets was comparable, with mean values of 4.44 and 4.29 and standard deviations of 0.68 and 0.70 for the training and test sets, respectively. As illustrated in the boxplot (Figure 1), both subsets covered a similar range of inhibitory potency, confirming the absence of systematic bias and supporting the representativeness of the dataset split.

The biomimetic chromatographic descriptors also exhibited significant variability across the dataset. The CHI IAM values ranged from −10.28 to 62.02 (mean 35.77 ± 14.39), reflecting a wide spectrum of phospholipid affinity, from weak to strong membrane interactions. Similarly, log *k*_HSA_ values spanned from −1.48 to 1.88 (mean 0.97 ± 0.64), indicating substantial differences in plasma protein binding among the compounds.

Inspection of the descriptor limits further supported the dataset's chemical plausibility. The lowest CHI IAM values were observed for isoniazid and salicylic acid, consistent with their small size, polarity, and/or ionizable character and reduced membrane-partitioning tendency. Conversely, the highest CHI IAM value was observed for amiodarone, followed by imatinib, atorvastatin, fluoxetine, and miconazole, which are characterized by greater lipophilicity, aromaticity, and structural complexity [14,16,17]. For log *k*_HSA_, the lowest values were observed for isoniazid, caffeine, sulpiride, erythromycin, and pravastatin, reflecting comparatively weaker albumin-binding propensity. The highest log *k*_HSA_ value was observed for benzbromarone, followed by ibuprofen, diclofenac, amiodarone, miconazole, and sulfonylureas, including glibenclamide/glyburide and glimepiride. These compounds are generally aromatic, lipophilic, and/or acidic/anionizable, consistent with stronger interactions with HSA [15,20]. These trends indicate that the boundary values of the biomimetic descriptors correspond to chemically interpretable structural features rather than arbitrary outliers.

The broad distribution of both the response variable and the selected descriptors ensure adequate coverage of chemical and physicochemical space, which is essential for developing robust, predictive QSAR models. Overall, the dataset was considered well-suited for modelling BSEP inhibition. No extreme skewness or abnormal distribution patterns were observed that would compromise model stability.

### Quantitative structure-activity relationship model development and comparison

A series of QSAR models was developed to evaluate the predictive performance of biomimetic chromatographic descriptors in comparison with conventional physicochemical parameters. The results are summarized in Table 1.

**Table 1.** Summary of developed QSAR models based on the training set (*n*=50) for the prediction of BSEP inhibition. Models based on biomimetic chromatographic descriptors (CHI IAM, log *k*_HSA_) are compared with conventional physicochemical models (log *P*, log *D*_7.4_). R: correlation coefficient; *R*^2^: coefficient of determination; *R*^2^_adj_: adjusted coefficient of determination; s: standard error of estimate; *F*: Fisher statistic

Model	Equation	*R*	*R* ^2^	*R* ^2^_adj_	*s*	*F*
M1 [CHI IAM]	pBSEP = 2.947 + 0.040·CHI IAM	0.838	0.702	0.696	0.372	113
M2 [CHI IAM + log *k*_HSA_]	pBSEP = 2.902 + 0.034·CHI IAM + 0.277·log *k*_HSA_	0.865	0.748	0.737	0.346	69.7
M3 [CHI IAM + log *k*_HSA_ + MW]	pBSEP = 2.646 + 0.029·CHI IAM + + 0.297·log *k*_HSA_ + 0.001·MW	0.898	0.806	0.794	0.307	63.8
M4 [log *P*]	pBSEP = 3.524 + 0.250·log *P*	0.599	0.359	0.346	0.546	26.9
M5 [log *P* + MW]	pBSEP = 3.112 + 0.195·log *P* + 0.001·MW	0.692	0.479	0.457	0.498	21.6
M6 [log *D*_7.4_]	pBSEP = 3.971 + 0.209·log *D*_7.4_	0.583	0.340	0.326	0.554	24.7
M7 [log *D*_7.4_ + MW]	pBSEP = 3.452 + 0.161·log *D*_7.4_ + 0.002·MW	0.682	0.465	0.442	0.504	20.4

The model based solely on the phospholipid affinity descriptor CHI IAM (M1) already demonstrated strong predictive ability (*R*^2^ = 0.702), indicating that membrane partitioning plays a significant role in BSEP inhibition. The positive coefficient of CHI IAM suggests that compounds with higher phospholipid affinity exhibit increased inhibitory potency, consistent with enhanced accumulation in membrane environments where BSEP is located.

Incorporating plasma protein binding *via* log *k*_HSA_ (M2) led to a notable improvement in model performance (*R*^2^ = 0.748), highlighting the additional contribution of protein binding to BSEP inhibition. The positive contribution of log *k*_HSA_ suggests that compounds with higher affinity for serum albumin tend to exhibit increased inhibitory potential, likely reflecting the interplay between binding, distribution, and effective exposure at the hepatocellular membrane. The log *k*_HSA_ should not be interpreted as a causal determinant of BSEP inhibition, but rather as an experimentally derived surrogate descriptor that captures systemic distribution characteristics that covary with membrane exposure and transporter interactions.

Further inclusion of molecular weight (MW) in the hybrid model (M3) yielded the highest predictive performance (*R*^2^ = 0.806), suggesting that size-related effects may also influence BSEP inhibition, potentially through steric or permeability-related mechanisms. However, the relatively modest improvement compared to M2 suggests that the primary determinants of BSEP inhibition are captured by the biomimetic descriptors.

In contrast, models based on conventional lipophilicity descriptors showed substantially lower predictive performance. The log *P*-based model (M4) yielded *R*^2^ = 0.359, while the log *D*_7.4_ model (M6) showed similar performance (*R*^2^ = 0.340), indicating that traditional bulk lipophilicity descriptors alone are insufficient to describe BSEP inhibition. The inclusion of molecular weight slightly improved these models (M5 and M7), but their performance remained significantly inferior to that of the biomimetic models.

The weaker performance of the conventional lipophilicity-based models may partly reflect the phenomenological limitations of log *P* and log *D*_7.4_. Although log *P* is widely used in QSAR modelling, it describes the partitioning behaviour of the neutral form of a compound and does not account for ionization at physiological pH. Since many drugs are partially or fully ionized under biological conditions, log *P* may not accurately reflect their in vivo distribution properties. Log *D*_7.4_ partially addresses this limitation by incorporating the apparent distribution of ionized and unionized species at physiological pH; however, it remains a simplified bulk descriptor. It does not explicitly capture microenvironmental interactions such as phospholipid affinity, membrane-specific partitioning, or plasma protein binding. These interactions may be influenced by ionization state, charge distribution, aromaticity, and molecular structure [14-16]. In contrast, biomimetic chromatographic descriptors inherently reflect experimentally observable interactions with membrane-like and protein-binding environments, contributing to their improved predictive performance and mechanistic relevance in the present study [14-17].

These findings highlight a key limitation of conventional descriptors, which primarily reflect bulk partitioning properties but fail to capture microenvironmental distribution phenomena, such as membrane affinity and protein binding [14-16]. In contrast, CHI IAM and log *k*_HSA_ provide experimentally derived proxies for membrane partitioning and plasma protein binding, respectively, enabling a more mechanistically relevant description of compound behaviour in biological systems.

The superior performance of biomimetic models demonstrates that experimentally derived chromatographic descriptors can significantly improve predictions of BSEP inhibition. The results support the hypothesis that local exposure at the membrane interface, rather than bulk physicochemical properties alone, is a critical determinant of transporter inhibition. This is consistent with the broader view that transporter-mediated toxicity is governed by the interplay between intrinsic potency and effective exposure at the site of interaction [1,4].

### Model validation and predictive performance

The predictive performance of the developed QSAR models was evaluated using both internal (Q^2^_cv_, RMSECV) and external (Q^2^_ext_, RMSEP) validation metrics (Table 2).

**Table 2.** Internal (training set; *n* = 50) and external (test set; *n* = 12) validation metrics of selected QSAR models for BSEP inhibition. *Q*^2^_cv_: cross-validated predictive coefficient; *Q*^2^_ext_: external predictive coefficient; RMSECV: root-mean-square error of cross-validation; RMSEP: root-mean-square error of prediction

Model	*R* ^2^	*Q* ^2^_cv_	*Q* ^2^_ext_	RMSECV	RMSEP
Phospholipid Binding (M1: CHI IAM)	0.702	0.671	0.664	0.384	0.398
Biomimetic (M2: CHI IAM + log *k*_HSA_)	0.748	0.696	0.667	0.369	0.370
Hybrid (M3: CHI IAM + log *k*_HSA_ + MW)	0.806	0.742	0.578	0.340	0.435
Conventional (M5: log *P* + MW)	0.479	0.327	0.588	0.549	0.440
Conventional (M7: log *D*_7.4_ + MW)	0.465	0.278	0.500	0.568	0.488

The biomimetic model based on CHI IAM and log *k*_HSA_ demonstrated strong and well-balanced performance, with *R*^2^ = 0.748, *Q*^2^_cv_ = 0.696, and *Q*^2^_ext_ = 0.667. The close agreement between internal and external validation metrics indicates good model stability and generalizability, suggesting that the model is not overfitted and retains predictive power for unseen compounds.

To further evaluate model parsimony, the predictive performance of the IAM-only model (M1) was compared with that of the two-descriptor biomimetic model (M2). The CHI IAM descriptor alone provided strong predictive capability, with *R*^2^ = 0.702, *Q*^2^_cv_ = 0.671, *Q*^2^_ext_ = 0.664, RMSECV = 0.384, and RMSEP = 0.398, indicating that membrane affinity represents the dominant biomimetic determinant of BSEP inhibition in the present dataset. Addition of log *k*_HSA_ modestly improved the training and internal validation statistics, increasing *R*^2^ to 0.748 and *Q*^2^_cv_ to 0.696, with a slightly lower RMSECV of 0.369. However, this improvement did not translate into a substantial gain in external predictive performance, as M2 showed a very similar *Q*^2^_ext_ value of 0.667 and RMSEP of 0.370. These results suggest that the IAM-only model may represent the most practical first-tier screening approach, particularly when minimizing experimental time and cost is important. The combined IAM and HSA model remains mechanistically informative because it integrates membrane affinity with albumin-binding propensity, but its use should be justified by the need for broader biomimetic characterization rather than by a clear improvement in external prediction. This comparison highlights the trade-off between experimental simplicity and mechanistic coverage in chromatography-based modelling of BSEP inhibition.

The hybrid model, which additionally incorporates molecular weight, achieved the highest goodness-of-fit (*R*^2^ = 0.806) and internal predictive performance (*Q*^2^_cv_ = 0.742). However, its external predictive ability was lower (*Q*^2^_ext_ = 0.578, RMSEP = 0.435), indicating reduced generalizability relative to the simpler biomimetic model. This behaviour suggests that including molecular weight, although it improves model fit, may introduce overfitting or capture dataset-specific variance that does not generalize to external compounds.

In contrast, models based on conventional physicochemical descriptors showed weaker internal predictive performance, with *Q*^2^_cv_ values of 0.327 for the log *P*-based model and 0.278 for the log *D*_7.4_ model. Although the log *P*-based model exhibited moderate external predictivity (*Q*^2^_ext_ = 0.588), the discrepancy between internal and external validation metrics indicates limited robustness and reduced reliability. The log *D*_7.4_-based model showed consistently lower performance across both internal and external validation metrics.

Among the multi-descriptor models, the biomimetic model provided the best balance among goodness-of-fit, robustness, interpretability, and external predictive performance. At the same time, the IAM-only model showed comparable external predictive performance, supporting its potential as a simpler first-tier screening tool. These findings highlight the advantage of experimentally derived chromatographic descriptors in capturing mechanistically relevant aspects of compound distribution, such as membrane affinity and protein binding, which are not adequately described by conventional lipophilicity parameters [14]. Most published models for BSEP inhibition are based on classification approaches and report metrics such as AUC and accuracy rather than continuous predictive performance [9-12]. In this context, the present model provides a quantitative prediction of inhibitory potency (*Q*^2^_ext_ = 0.667) and offers enhanced mechanistic interpretability through biomimetic descriptors.

Importantly, the results support the concept that BSEP inhibition is not solely governed by intrinsic potency or bulk physicochemical properties but is strongly driven by membrane-proximal exposure [1,4]. By incorporating experimentally derived descriptors that reflect these processes, the biomimetic model offers improved predictive performance and mechanistic interpretability compared to traditional QSAR approaches [14-16].

To further assess the robustness and reliability of the selected biomimetic model, graphical validation techniques and applicability domain analysis were performed.

**Figure 2.**
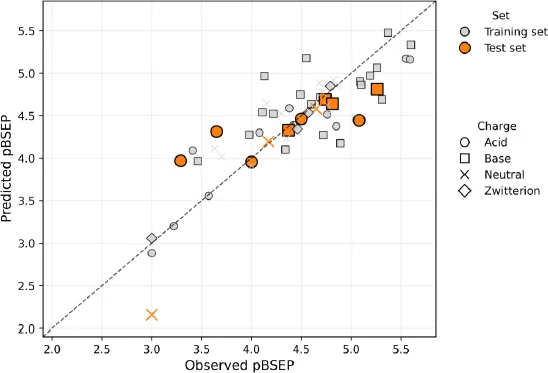
Observed *versus* predicted pBSEP values for the IAM + HSA biomimetic model. The dashed line represents the line of identity, indicating perfect agreement between experimental and predicted values. Training set compounds are shown in grey, while external test set compounds are highlighted in orange. Marker shapes indicate compound charge class

The observed-versus-predicted plot for the biomimetic model (Figure 2) demonstrates strong agreement between experimental and predicted pBSEP values for both the training and external test sets. Most data points are clustered near the identity line, indicating that the model accurately captures the relationship between the selected descriptors and BSEP inhibition.

The training-set compounds are tightly clustered around the diagonal, reflecting good model fit and consistency across the calibration dataset. Importantly, the test set compounds also follow the same trend, confirming the model’s ability to generalize to unseen data and supporting its external predictive performance. Although a small number of compounds deviate slightly from the ideal line, no systematic overestimation or underestimation is observed across the range of pBSEP values. The deviations appear randomly distributed, suggesting that prediction errors are not associated with specific regions of the response space. Furthermore, the model maintains good predictive performance across compounds with different ionization states, as indicated by the distribution of acids, bases, and neutral molecules in the plot. This suggests that the biomimetic descriptors effectively capture underlying distributional properties relevant across diverse chemical classes. The absence of systematic deviation at higher or lower pBSEP values further indicates that the model does not suffer from range-dependent bias.

**Figure 3.**
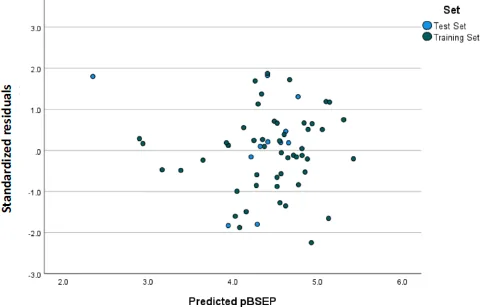
Standardized residuals *versus* predicted pBSEP values for the biomimetic model (CHI IAM + log *k*_HSA_) for both training and test sets

Residual analysis (Figure 3) shows that standardized residuals are randomly distributed around zero across the full range of predicted pBSEP values. No systematic patterns, trends, or curvature are observed, indicating that the assumptions of linearity and homoscedasticity are satisfied.

The residuals are symmetrically distributed around the zero line, with most values falling within the acceptable range (|*r*ᵢ| ≤ 2), and all values remaining within the critical threshold (|*r*ᵢ| ≤ 3). This suggests the absence of extreme outliers and confirms the model's stability. Importantly, no increase in residual variance is observed for higher or lower predicted values, indicating that the model does not exhibit heteroscedasticity. The consistent spread of residuals across the prediction range supports the model's reliability for both weak and strong inhibitors.

**Figure 4.**
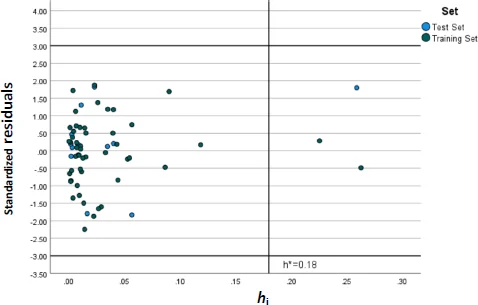
Williams plot for the biomimetic model (CHI IAM + log *k*_HSA_), showing standardized residuals *versus* leverage values (*h*ᵢ). The horizontal lines represent the ±3 residual limits, and the vertical line indicates the warning leverage threshold (h* = 0.18)

The applicability domain of the biomimetic model was evaluated using the Williams plot (Figure 4), where standardized residuals were plotted against leverage values. Most compounds are located within the defined boundaries (|*r*ᵢ| ≤ 3 and *h*ᵢ ≤ h* = 0.18), indicating that predictions for most compounds are reliable and fall within the model’s domain.

A small number of compounds, namely isoniazid, ibuprofen, and salicylic acid, exhibited leverage values exceeding the warning threshold (*h*ᵢ > h*), indicating that they occupy influential regions of the descriptor space. However, their standardized residuals remained within acceptable limits (|*r*ᵢ| ≤ 3), suggesting that they are not response outliers and that their predicted values remain consistent with the model [19]. The high leverage observed for ibuprofen and salicylic acid can be rationalized based on their structural and physicochemical characteristics. Both compounds are relatively small aromatic carboxylic acids within the acidic NSAID/salicylate-like chemical space.

Their ionizable acidic functionality, combined with aromatic character and appreciable albumin-binding propensity, gives them a distinctive biomimetic profile [15,20]. In particular, these compounds combine comparatively low-to-moderate IAM affinity with relatively high HSA interaction, positioning them at the edge of the descriptor space relative to the broader dataset [14-16]. Other NSAIDs present in the dataset, such as diclofenac, indomethacin, etodolac, and sulindac, exhibit similar acidic/aromatic features but do not exceed the leverage threshold because their IAM and HSA descriptor values lie closer to the model's main chemical space. Therefore, ibuprofen and salicylic acid should be interpreted as edge cases within the acidic NSAID-like region rather than as evidence of systematic class-wide outlier behaviour. Importantly, no compounds exceeded both the leverage and residual thresholds simultaneously, confirming the absence of influential outliers and supporting the model's robustness within its applicability domain [19].

To assess the possibility of chance correlation, Y-randomization tests were performed by randomly permuting the response variable while keeping the descriptor matrix unchanged. The models generated from randomized data exhibited significantly lower *R*^2^ and *Q*^2^ values than the original model (Table 3).

**Table 3.** Y-randomization results for the biomimetic model. *R*^2^ and *Q*^2^ values for models generated from randomized response data are compared with those of the original model

Model	*R* ^2^	*Q* ^2^
Original	0.748	0.696
Random 1	0.12	-0.05
Random 2	0.08	-0.12

In particular, the randomized models showed very low *R*^2^ values (≤ 0.12) and negative or near-zero *Q*^2^ values, indicating a complete loss of predictive ability. In contrast, the original biomimetic model demonstrated substantially higher performance (*R*^2^ = 0.748, *Q*^2^ = 0.696), confirming that the observed correlation is not due to random effects. These results strongly support the statistical robustness of the developed QSAR model and confirm that its predictive performance arises from meaningful relationships between descriptors and response rather than chance correlation.

### Mechanistic interpretation

The superior performance of the biomimetic model can be interpreted in the context of BSEP biology and the distributional behaviour of drug molecules. BSEP is an ATP-dependent efflux transporter located at the canalicular membrane of hepatocytes and represents the rate-limiting step in bile acid secretion. Because inhibition occurs at a membrane-embedded target, local exposure at the membrane interface is likely to be more relevant than bulk physicochemical properties alone. The International Transporter Consortium has emphasized that the interpretation of BSEP inhibition should take into account *in vivo* exposure and local concentrations rather than relying solely on in vitro inhibitory potency [1,4].

The positive contribution of CHI IAM is mechanistically consistent with this view. IAM chromatography is widely used as an experimental surrogate for phospholipid affinity and membrane partitioning, and recent reviews describe IAM retention as informative for permeability, tissue binding, and off-target binding processes [14-16]. Thus, higher CHI IAM values can reasonably be interpreted as indicating a greater tendency of a compound to partition into membrane-like environments, potentially favouring local enrichment near the canalicular membrane, where BSEP resides.

This interpretation is also supported by the BSEP literature, which shows that inhibition is strongly associated with lipophilicity. Pedersen et al. reported that BSEP inhibition correlates strongly with compound lipophilicity, whereas positive molecular charge is associated with reduced inhibition. They further note that transported BSEP substrates are preferably monovalent, negatively charged bile acids, and the few known non-bile acid substrates also carry a negative net charge at physiological pH [7]. Taken together, these observations support the idea that membrane-affine, non-cationic compounds are more likely to occupy the physicochemical space compatible with BSEP interaction.

**Figure 5.**
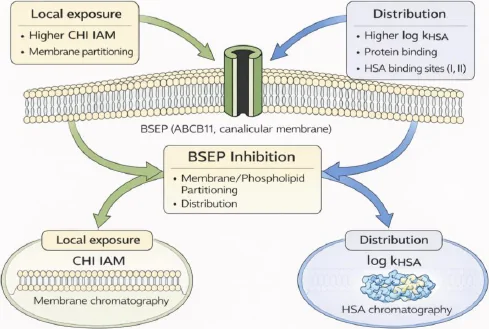
Mechanistic framework linking biomimetic chromatographic descriptors to BSEP inhibition. CHI IAM reflects phospholipid affinity and membrane partitioning, while log *k*_HSA_ captures protein-associated distribution and albumin binding. Together, these descriptors approximate local exposure at the canalicular membrane, providing a mechanistic basis for predicting BSEP inhibition

The inclusion of log *k*_HSA_ further improved model performance, although its role requires careful interpretation. Albumin binding itself is not a direct mechanistic driver of BSEP inhibition; rather, log *k*_HSA_ should be viewed as an experimentally derived descriptor of a broader distributional phenotype [15]. Compounds with higher albumin affinity often share physicochemical features, such as hydrophobicity and anionic character, that are also associated with hepatobiliary distribution and transporter interaction. Human serum albumin is the major plasma carrier protein and binds a wide range of drugs, thereby limiting their free concentrations and modulating systemic transport and distribution [15]. Structural and biochemical studies show that HSA contains major drug-binding pockets, including the Sudlow sites I and II; site II preferentially binds aromatic compounds such as ibuprofen, whereas site I binds ligands such as warfarin [20]. These complementary mechanistic roles of CHI IAM and log *k*_HSA_ are schematically summarized in Figure 5.

More generally, HSA is recognized as an important carrier for many acidic drugs [15,20]. In this sense, log *k*_HSA_ complements CHI IAM by capturing systemic distribution behaviour that covaries with membrane exposure. The combination of these descriptors therefore provides a more complete representation of microenvironmental exposure conditions relevant to BSEP interaction than conventional descriptors such as log *P* or log *D*_7.4_ alone [1,4,14].

The present findings support a mechanistically plausible framework in which BSEP inhibition is favoured by compounds that combine appreciable membrane affinity with distribution characteristics typical of albumin-binding. While these descriptors do not replace transporter assays, they provide experimentally grounded proxies for the physicochemical and distributional determinants that govern BSEP interactions, thereby supporting their use in early-stage risk assessment. This framework may therefore support early prioritization of compounds with reduced cholestatic liability during drug discovery.

## Conclusions

The present study introduces a chromatography-informed modelling framework for predicting BSEP inhibition using experimentally derived descriptors of membrane affinity and protein-associated distribution. The results show that biomimetic chromatographic descriptors, particularly CHI IAM and log *k*_HSA_, provide an interpretable and experimentally accessible way to capture distribution-related determinants of BSEP inhibition that are not fully represented by conventional physicochemical parameters such as log *P* and log *D*_7.4_.

A central finding of this work is that membrane affinity, represented by CHI IAM, is a dominant determinant of BSEP inhibition in the present dataset. The IAM-only model showed strong predictive performance and external predictivity comparable to the two-descriptor biomimetic model, supporting its potential use as a practical first-tier screening approach when experimental simplicity and throughput are priorities. The addition of log *k*_HSA_ modestly improved training and internal cross-validation performance and provided complementary mechanistic information on albumin-binding propensity, highlighting a practical trade-off between experimental simplicity and broader biomimetic characterization.

The improved performance of biomimetic models relative to conventional log *P*/log *D*-based models supports the hypothesis that BSEP inhibitory liability is not adequately described by bulk lipophilicity alone but is also influenced by distribution-related factors that determine compound enrichment and interaction at the membrane interface. This is particularly relevant for BSEP, which is localized at the canalicular membrane, where membrane-proximal compound enrichment and protein-associated distribution may influence the effective concentration available for transporter interaction. Conventional lipophilicity descriptors remain useful reference parameters, but they cannot fully account for ionization-dependent membrane interactions, phospholipid affinity, or protein binding.

Importantly, the proposed model should not be interpreted as a direct predictor of clinical DILI. Clinical DILI is a multifactorial outcome influenced by metabolic processes, mitochondrial toxicity, immune-mediated mechanisms, dose and exposure, and patient-specific susceptibility. Instead, the present approach is best positioned as a mechanistically informed screening layer for identifying compounds with potential BSEP-mediated cholestatic liability. It can complement, but not replace, in vitro transporter assays, hepatocyte-based systems, and integrated DILI risk-assessment frameworks.

This study demonstrates the value of biomimetic chromatography as a bridge between physicochemical characterization and mechanistic toxicology. By linking experimentally measured membrane and protein-binding interactions to transporter inhibition, the proposed framework provides a practical, interpretable, and scalable tool for early compound prioritization. Future work should focus on expanding the dataset's chemical space, validating the approach across larger external compound sets, incorporating additional mechanistic descriptors related to intracellular accumulation and metabolism, and integrating biomimetic models into multi-parameter DILI risk-assessment strategies.

## Supplementary material


