# Supplementary material for: Prediction of bile salt export pump inhibition using biomimetic chromatographic descriptors: Integrating membrane affinity and protein binding
Source: ADMET DMPK. 2026 May 24;14:3303. doi: 10.5599/admet.3303 (PMC13499664; doi:10.5599/admet.3303)
Supplement: Supplementary file 1 [file ADMET-14-3303-S1.pdf]

ADMET & DMPK **14** (2026) S3303

Open Access : ISSN : 1848-7718
http://www.pub.iapchem.org/ojs/index.php/admet

Supplementary material to

Prediction of bile salt export pump inhibition using biomimetic chromatographic descriptors: Integrating membrane affinity and protein binding

Chrysanthos Stergiopoulos[1] and Klara Valko[2]

[1]*Laboratory of Process Analysis and Design, School of Chemical Engineering, National Technical University of Athens, Iroon Polytechniou 9, 157 75 Zografou, Athens, Greece*
[2]*Bio-Mimetic Chromatography Ltd, Business & Technology Centre, Bessemer Drive, Stevenage, Herts, SG1 2DX, United Kingdom*

ADMET & DMPK **14** (2026) 3303; https://doi.org/10.5599/admet.3303

Table S1. Reported *in vitro* BSEP inhibition dataset used in the present study, expressed as pBSEP values (−log (IC_{50}, M)) [1-4], together with the assignment of compounds to the training set (n = 50) and external test set (n = 12).

No.	Compound	pBSEP	Set
1	17-a-Ethinyl-Estradiol	4.85	Training
2	Acitretin	4.42	
3	Amiodarone	5.37	
4	Amprenavir	4.35	
5	Atorvastatin	5.59	
6	Benzbromarone	5.55	
7	Bezafibrate	3.41	
8	Bicalutamide	4.65	
9	Buspirone	4.72	
10	Caffeine	3.70	
11	Chlorpromazine	5.19	
12	Clofibrate	4.15	
13	Dapsone	4.00	
14	Dexamethasone	3.63	
15	Donepezil	4.11	
16	Erythromycin	4.89	
17	Etodolac	4.08	
18	Fenofibrate	4.82	
19	Fluoxetine	5.26	
20	Furosemide	4.00	
21	Fusidic acid	4.76	
22	Gefitinib	5.09	
23	Glimepiride	4.85	
24	Glyburide	5.10	
25	Haloperidol	4.60	
26	Ibuprofen	3.22	
27	Imatinib	5.60	
28	Imipramine	4.22	
29	Indomethacin	4.38	
30	Leflunomide	4.30	
31	Lopinavir	4.76	
32	Lovastatin	4.71	
33	Miconazole	4.55	

No.	Compound	pBSEP	Set
34	Nicardipine	5.10	Training
35	Nifedipine	4.34	
36	Paclitaxel	4.78	
37	Papaverine	3.98	
38	Paracetamol	3.00	
39	Pinacidil	3.46	
40	Pravastatin	3.57	
41	Primaquine	4.49	
42	Rifabutin	4.57	
43	Rifampicin	4.46	
44	Salicylic acid	3.00	
45	Saquinavir	5.31	
46	Simvastatin	4.68	
47	Sulpiride	3.00	
48	Telmisartan	4.79	
49	Ticlopidine	4.13	
50	Verapamil	4.69	
51	Clotrimazole	5.26	Test
52	Diclofenac	4.50	
53	Glibenclamide	5.08	
54	Isoniazid	3.00	
55	Itraconazole	4.74	
56	Ketoconazole	4.81	
57	Nitrendipine	4.65	
58	Omeprazole	4.00	
59	Praziquantel	4.17	
60	Probenecid	3.29	
61	Sulindac	3.65	
62	Trazodone	4.37	

Table S2. Experimentally determined phospholipid affinity descriptors expressed as CHI IAM values for the 62 compounds included in the dataset. CHI IAM was used as a biomimetic chromatographic descriptor of membrane/phospholipid partitioning

No.	Compound	CHI IAM
1	17-a-Ethinyl-Estradiol	45.1
2	Acitretin	33.2
3	Amiodarone	62.0
4	Amprenavir	32.4
5	Atorvastatin	56.5
6	Benzbromarone	50.8
7	Bezafibrate	25.0
8	Bicalutamide	37.8
9	Buspirone	34.8
10	Caffeine	3.18
11	Chlorpromazine	48.9
12	Clofibrate	40.5
13	Clotrimazole	43.5
14	Dapsone	24.5
15	Dexamethasone	31.9
16	Diclofenac	34.2
17	Donepezil	41.5
18	Erythromycin	38.8
19	Etodolac	30.6
20	Fenofibrate	46.8
21	Fluoxetine	54.9
22	Furosemide	20.1

CC BY

No.	Compound	CHI IAM
23	Fusidic acid	36.2
24	Gefitinib	49.1
25	Glibenclamide	32.6
26	Glimepiride	30.8
27	Glyburide	32.6
28	Haloperidol	44.3
29	Ibuprofen	20.6
30	Imatinib	60.9
31	Imipramine	39.4
32	Indomethacin	30.1
33	Isoniazid	-10.3
34	Itraconazole	40.6
35	Ketoconazole	41.1
36	Leflunomide	39.0
37	Lopinavir	40.1
38	Lovastatin	44.4
39	Miconazole	53.7
40	Nicardipine	47.0
41	Nifedipine	25.1
42	Nitrendipine	39.4
43	Omeprazole	21.1
44	Paclitaxel	39.2
45	Papaverine	32.0
46	Paracetamol	2.69
47	Pinacidil	29.7
48	Pravastatin	20.5
49	Praziquantel	31.5
50	Primaquine	49.2
51	Probenecid	19.8
52	Rifabutin	43.9
53	Rifampicin	34.7
54	Salicylic acid	-8.67
55	Saquinavir	42.2
56	Simvastatin	46.8
57	Sulindac	30.3
58	Sulpiride	7.35
59	Telmisartan	45.6
60	Ticlopidine	48.3
61	Trazodone	32.7
62	Verapamil	44.8

Table S3. Experimentally determined human serum albumin affinity descriptors expressed as log k_{HSA} values for the 62 compounds included in the dataset. log k_{HSA} was used as a biomimetic chromatographic descriptor of plasma protein binding.

No.	Compound	log k_{HSA}
1	17-a-Ethinyl-Estradiol	1.45
2	Acitretin	1.25
3	Amiodarone	1.59
4	Amprenavir	0.78
5	Atorvastatin	1.14
6	Benzbromarone	1.88
7	Bezafibrate	1.19
8	Bicalutamide	1.36
9	Buspirone	0.62
10	Caffeine	-0.56
11	Chlorpromazine	1.35

No.	Compound	log k_{HSA}
12	Clofibrate	1.27
13	Clotrimazole	1.49
14	Dapsone	0.60
15	Dexamethasone	0.41
16	Diclofenac	1.73
17	Donepezil	0.76
18	Erythromycin	-0.22
19	Etodolac	1.25
20	Fenofibrate	1.46
21	Fluoxetine	0.99
22	Furosemide	1.24
23	Fusidic acid	1.32
24	Gefitinib	1.13
25	Glibenclamide	1.52
26	Glimepiride	1.50
27	Glyburide	1.52
28	Haloperidol	0.76
29	Ibuprofen	1.84
30	Imatinib	1.22
31	Imipramine	0.95
32	Indomethacin	1.21
33	Isoniazid	-1.48
34	Itraconazole	1.42
35	Ketoconazole	1.18
36	Leflunomide	1.02
37	Lopinavir	1.25
38	Lovastatin	1.24
39	Miconazole	1.54
40	Nicardipine	1.25
41	Nifedipine	0.67
42	Nitrendipine	1.17
43	Omeprazole	1.18
44	Paclitaxel	0.99
45	Papaverine	0.99
46	Paracetamol	0.21
47	Pinacidil	0.14
48	Pravastatin	-0.18
49	Praziquantel	0.76
50	Primaquine	0.56
51	Probenecid	1.39
52	Rifabutin	0.46
53	Rifampicin	0.89
54	Salicylic acid	1.01
55	Saquinavir	1.22
56	Simvastatin	1.34
57	Sulindac	1.34
58	Sulpiride	-0.35
59	Telmisartan	1.36
60	Ticlopidine	1.45
61	Trazodone	1.09
62	Verapamil	1.00

Table S4. Calculated physicochemical descriptors for the compounds included in the dataset, obtained using the ACD/Percepta Platform. Descriptors include lipophilicity (log *P*), distribution coefficient at pH 7.4 (log $D_{7.4}$), molecular weight (MW), hydrogen-bond donors (HBD), hydrogen-bond acceptors (HBA), topological polar surface area (TPSA), Abraham descriptors (A and B), fractional charge descriptors (fpos: fraction positively charged, fneg: fraction negatively charged, fzw: fraction zwitterionic species) and overall ionization class

Compound	log *P*	log $D_{7.4}$	MW	HBD	HBA	TPSA, nm^2	A	B	fpos	fneg	fzw	Charge
17-a-Ethinyl-Estradiol	3.67	3.98	296	2.00	2.00	40.5	0.90	1.02	0.00	0.00	0.00	Neutral
Acitretin	6.4	3.14	326	1.00	3.00	46.5	0.57	0.97	0.00	1.00	0.00	Acid
Amiodarone	7.80	6.38	645	0.00	4.00	42.7	0.00	1.30	0.96	0.00	0.00	Base
Amprenavir	3.94	3.94	506	4.00	9.00	140	0.64	2.61	0.00	0.00	0.00	Neutral
Atorvastatin	5.19	2.22	559	4.00	7.00	112	1.61	2.07	0.00	1.00	0.00	Acid
Benzbromarone	5.74	2.78	424	1.00	3.00	50.4	0.41	0.68	0.00	1.00	0.00	Acid
Bezafibrate	3.98	0.66	362	2.00	5.00	75.6	0.83	1.35	0.00	1.00	0.00	Acid
Bicalutamide	2.93	2.93	430	2.00	6.00	116	0.71	1.63	0.00	0.00	0.00	Neutral
Buspirone	2.63	1.77	386	0.00	7.00	69.6	0.00	2.16	0.64	0.00	0.00	Weak Base
Caffeine	-0.07	-0.45	194	0.00	6.00	58.4	0.05	1.28	0.00	0.00	0.00	Neutral
Chlorpromazine	5.35	3.34	319	0.00	2.00	31.8	0.00	0.94	0.99	0.00	0.00	Base
Clofibrate	3.52	3.52	243	0.00	3.00	35.5	0.00	0.69	0.00	0.00	0.00	Neutral
Clotrimazole	4.99	5.07	345	0.00	2.00	17.8	0.00	0.83	0.58	0.00	0.00	Weak Base
Dapsone	0.97	1.42	248	4.00	4.00	94.6	0.45	1.35	0.00	0.00	0.00	Neutral
Dexamethasone	1.83	1.80	392	3.00	5.00	94.8	0.71	1.92	0.00	0.00	0.00	Neutral
Diclofenac	4.40	1.23	296	2.00	3.00	49.3	0.63	0.96	0.00	1.00	0.00	Acid
Donepezil	3.75	2.05	379	0.00	4.00	38.8	0.00	1.50	0.98	0.00	0.00	Base
Erythromycin	2.54	0.65	734	5.00	14.00	194	1.05	4.63	0.95	0.00	0.00	Base
Etodolac	3.81	-0.05	287	2.00	4.00	62.3	0.88	0.90	0.00	1.00	0.00	Acid
Fenofibrate	4.38	4.38	361	0.00	4.00	52.6	0.00	1.13	0.00	0.00	0.00	Neutral
Fluoxetine	4.50	2.44	309	1.00	2.00	21.3	0.13	0.78	1.00	0.00	0.00	Base
Furosemide	2.03	-1.59	331	4.00	7.00	131	1.35	1.45	0.00	1.00	0.00	Acid
Fusidic acid	5.56	2.78	517	3.00	6.00	104	1.20	1.88	0.00	1.00	0.00	Acid
Gefitinib	4.76	4.46	447	1.00	7.00	68.7	0.25	1.87	0.53	0.00	0.00	Weak Base
Glibenclamide	4.02	1.83	494	3.00	8.00	122	0.85	2.01	0.00	0.99	0.00	Acid
Glimepiride	4.25	2.05	491	3.00	9.00	133	0.75	2.15	0.00	0.99	0.00	Acid
Glyburide	4.02	1.83	494	3.00	8.00	122	0.85	2.01	0.00	0.99	0.00	Acid
Haloperidol	3.36	2.18	376	1.00	3.00	40.5	0.40	1.76	0.99	0.00	0.00	Base
Ibuprofen	3.50	0.38	206	1.00	2.00	37.3	0.59	0.81	0.00	1.00	0.00	Acid
Imatinib	2.22	1.81	494	2.00	8.00	86.3	0.54	2.63	0.64	0.00	0.00	Weak Base
Imipramine	4.28	2.61	280	0.00	2.00	4.80	0.00	1.15	0.99	0.00	0.00	Base
Indomethacin	4.27	0.71	358	1.00	5.00	68.5	0.57	1.57	0.00	1.00	0.00	Acid
Isoniazid	-0.81	-0.81	137	3.00	4.00	68.0	0.47	1.39	0.00	0.00	0.00	Neutral
Itraconazole	5.66	4.32	706	0.00	12.00	101	0.00	2.95	0.01	0.00	0.00	Neutral
Ketoconazole	4.34	3.98	531	0.00	8.00	69.1	0.00	2.22	0.15	0.00	0.00	Weak Base
Leflunomide	1.59	1.59	270	1.00	4.00	55.1	0.47	0.81	0.00	0.00	0.00	Neutral
Lopinavir	5.16	5.16	629	4.00	9.00	120	0.92	2.89	0.00	0.00	0.00	Neutral
Lovastatin	4.26	4.40	405	1.00	5.00	72.8	0.31	1.44	0.00	0.00	0.00	Neutral
Miconazole	5.34	5.45	416	0.00	3.00	27.1	0.00	0.79	0.15	0.00	0.00	Weak Base
Nicardipine	4.65	2.66	480	1.00	9.00	117	0.13	2.12	0.41	0.00	0.00	Weak Base
Nifedipine	3.27	1.12	346	1.00	8.00	113	0.23	1.45	1.00	0.00	0.00	Base
Nitrendipine	4.15	1.6	360	1.00	8.00	113	0.13	1.54	0.00	0.00	0.00	Neutral
Omeprazole	2.23	2.37	345	1.00	6.00	96.3	0.35	2.05	0.00	0.05	0.00	Neutral
Paclitaxel	3.95	3.95	854	4.00	15.00	221	0.90	4.13	0.00	0.00	0.00	Neutral
Papaverine	2.95	3.65	339	0.00	5.00	49.8	0.00	1.47	0.07	0.00	0.00	Neutral
Paracetamol	0.46	0.23	151	2.00	3.00	49.3	1.04	0.86	0.00	0.00	0.00	Neutral
Pinacidil	2.87	2.85	245	2.00	5.00	73.1	0.39	1.54	0.04	0.00	0.00	Neutral
Pravastatin	2.18	-1.08	425	4.00	7.00	124	1.51	1.89	0.00	1.00	0.00	Acid
Praziquantel	3.02	3.02	312	0.00	4.00	40.6	0.00	1.46	0.00	0.00	0.00	Neutral
Primaquine	2.86	-0.34	259	3.00	4.00	60.2	0.34	1.49	1.00	0.00	0.00	Base
Probenecid	3.21	-0.22	285	1.00	5.00	83.1	0.57	1.29	0.00	1.00	0.00	Acid
Rifabutin	4.50	1.86	847	5.00	15.00	206	1.31	4.39	0.81	0.00	0.18	Weak Base
Rifampicin	2.39	1.68	823	6.00	16.00	220	2.55	4.66	0.64	0.00	0.14	Weak Base
Salicylic acid	2.26	-1.89	138	2.00	3.00	57.5	0.71	0.38	0.00	1.00	0.00	Acid
Saquinavir	3.77	3.67	671	6.00	11.00	167	1.46	3.89	0.22	0.00	0.00	Weak Base
Simvastatin	4.87	4.87	419	1.00	5.00	72.8	0.31	1.45	0.00	0.00	0.00	Neutral
Sulindac	3.42	0.26	356	1.00	3.00	73.6	0.57	1.39	0.00	1.00	0.00	Acid
Sulpiride	0.62	-1.28	341	3.00	7.00	110	0.72	2.15	0.98	0.01	0.00	Base
Telmisartan	7.44	4.06	515	1.00	6.00	72.9	0.60	1.95	0.00	0.93	0.07	Acid
Ticlopidine	3.68	3.38	264	0.00	1.00	31.5	0.00	0.62	0.53	0.00	0.00	Weak Base
Trazodone	3.80	4.60	372	0.00	6.00	42.4	0.00	1.92	0.22	0.00	0.00	Weak Base
Verapamil	3.83	3.17	455	0.00	6.00	64.0	0.00	1.89	0.98	0.00	0.00	Base

References

[1] S. Dawson, S. Stahl, N. Paul, J. Barber, J.G. Kenna. *In vitro* inhibition of the bile salt export pump correlates with risk of cholestatic drug-induced liver injury in humans. *Drug Metabolism and Disposition* **40** (2012) 130-138. https://dx.doi.org/10.1124/dmd.111.040758

[2] R.E. Morgan, C.J. van Staden, Y. Chen, N. Kalyanaraman, J. Kalanzi, R.T. Dunn, C.A. Afshari, H.K. Hamadeh. A multifactorial approach to hepatobiliary transporter assessment enables improved therapeutic compound development. *Toxicological Sciences* **136** (2013) 216-241. https://dx.doi.org/10.1093/toxsci/kft176

[3] J.M. Pedersen, P. Matsson, C.A. Bergström, J. Hoogstraate, A. Norén, E.L. LeCluyse, P. Artursson. Early identification of clinically relevant drug interactions with the human bile salt export pump (BSEP/ABCB11). *Toxicological Sciences* **136** (2013) 328-343. https://dx.doi.org/10.1093/toxsci/kft197

[4] F. Montanari, M. Pinto, N. Khunweeraphong, K. Wlcek, M.I. Sohail, T. Noeske, S. Boyer, P. Chiba, B. Stieger, K. Kuchler, G.F. Ecker. Flagging Drugs That Inhibit the Bile Salt Export Pump. *Molecular Pharmaceutics* **13** (2016) 163-171. https://dx.doi.org/10.1021/acs.molpharmaceut.5b00594

(cc) BY
