# Supplementary material for: Prediction of bile salt export pump inhibition using biomimetic chromatographic descriptors: Integrating membrane affinity and protein binding
Source: ADMET DMPK. 2026 May 24;14:3303. doi: 10.5599/admet.3303 (PMC13499664; doi:10.5599/admet.3303)
Supplement: Supplementary file 2 [file ADMET-14-3303-S1.docx]

ADMET & DMPK **14** (2026) S3303

**Open Access : ISSN : 1848-7718**
[**http://www.pub.iapchem.org/ojs/index.php/admet**](http://www.pub.iapchem.org/ojs/index.php/admet)

Supplementary material to

**Prediction of bile salt export pump inhibition using biomimetic chromatographic descriptors: Integrating membrane affinity and protein binding**

Chrysanthos Stergiopoulos^1^^<https://orcid.org/0000-0001-6002-4870>^ and Klara Valko^2^^<https://orcid.org/0000-0003-4605-2941>^

*^1^Laboratory of Process Analysis and Design, School of Chemical Engineering, National Technical University of Athens, Iroon Polytechniou 9, 157 75 Zografou, Athens, Greece
^2^Bio-Mimetic Chromatography Ltd, Business & Technology Centre, Bessemer Drive, Stevenage, Herts, SG1 2DX, United Kingdom*

ADMET & DMPK **14** (2026) 3303; <https://doi.org/10.5599/admet.3303>

**Table S1.** Reported *in vitro* BSEP inhibition dataset used in the present study, expressed as pBSEP values (−log (IC₅₀, M)) [1-4], together with the assignment of compounds to the training set (*n* = 50) and external test set (*n* = 12).

| No. | Compound | pBSEP | Set |
| --- | --- | --- | --- |
| 1 | 17-a-Ethinyl-Estradiol | 4.85 | Training |
| 2 | Acitretin | 4.42 |  |
| 3 | Amiodarone | 5.37 |  |
| 4 | Amprenavir | 4.35 |  |
| 5 | Atorvastatin | 5.59 |  |
| 6 | Benzbromarone | 5.55 |  |
| 7 | Bezafibrate | 3.41 |  |
| 8 | Bicalutamide | 4.65 |  |
| 9 | Buspirone | 4.72 |  |
| 10 | Caffeine | 3.70 |  |
| 11 | Chlorpromazine | 5.19 |  |
| 12 | Clofibrate | 4.15 |  |
| 13 | Dapsone | 4.00 |  |
| 14 | Dexamethasone | 3.63 |  |
| 15 | Donepezil | 4.11 |  |
| 16 | Erythromycin | 4.89 |  |
| 17 | Etodolac | 4.08 |  |
| 18 | Fenofibrate | 4.82 |  |
| 19 | Fluoxetine | 5.26 |  |
| 20 | Furosemide | 4.00 |  |
| 21 | Fusidic acid | 4.76 |  |
| 22 | Gefitinib | 5.09 |  |
| 23 | Glimepiride | 4.85 |  |
| 24 | Glyburide | 5.10 |  |
| 25 | Haloperidol | 4.60 |  |
| 26 | Ibuprofen | 3.22 |  |
| 27 | Imatinib | 5.60 |  |
| 28 | Imipramine | 4.22 |  |
| 29 | Indomethacin | 4.38 |  |
| 30 | Leflunomide | 4.30 |  |
| 31 | Lopinavir | 4.76 |  |
| 32 | Lovastatin | 4.71 |  |
| 33 | Miconazole | 4.55 |  |
| 34 | Nicardipine | 5.10 | Training |
| 35 | Nifedipine | 4.34 |  |
| 36 | Paclitaxel | 4.78 |  |
| 37 | Papaverine | 3.98 |  |
| 38 | Paracetamol | 3.00 |  |
| 39 | Pinacidil | 3.46 |  |
| 40 | Pravastatin | 3.57 |  |
| 41 | Primaquine | 4.49 |  |
| 42 | Rifabutin | 4.57 |  |
| 43 | Rifampicin | 4.46 |  |
| 44 | Salicylic acid | 3.00 |  |
| 45 | Saquinavir | 5.31 |  |
| 46 | Simvastatin | 4.68 |  |
| 47 | Sulpiride | 3.00 |  |
| 48 | Telmisartan | 4.79 |  |
| 49 | Ticlopidine | 4.13 |  |
| 50 | Verapamil | 4.69 |  |
| 51 | Clotrimazole | 5.26 | Test |
| 52 | Diclofenac | 4.50 |  |
| 53 | Glibenclamide | 5.08 |  |
| 54 | Isoniazid | 3.00 |  |
| 55 | Itraconazole | 4.74 |  |
| 56 | Ketoconazole | 4.81 |  |
| 57 | Nitrendipine | 4.65 |  |
| 58 | Omeprazole | 4.00 |  |
| 59 | Praziquantel | 4.17 |  |
| 60 | Probenecid | 3.29 |  |
| 61 | Sulindac | 3.65 |  |
| 62 | Trazodone | 4.37 |  |

**Table S2.** Experimentally determined phospholipid affinity descriptors expressed as CHI IAM values for the 62 compounds included in the dataset. CHI IAM was used as a biomimetic chromatographic descriptor of membrane/phospholipid partitioning

| No. | Compound | CHI IAM |
| --- | --- | --- |
| 1 | 17-a-Ethinyl-Estradiol | 45.1 |
| 2 | Acitretin | 33.2 |
| 3 | Amiodarone | 62.0 |
| 4 | Amprenavir | 32.4 |
| 5 | Atorvastatin | 56.5 |
| 6 | Benzbromarone | 50.8 |
| 7 | Bezafibrate | 25.0 |
| 8 | Bicalutamide | 37.8 |
| 9 | Buspirone | 34.8 |
| 10 | Caffeine | 3.18 |
| 11 | Chlorpromazine | 48.9 |
| 12 | Clofibrate | 40.5 |
| 13 | Clotrimazole | 43.5 |
| 14 | Dapsone | 24.5 |
| 15 | Dexamethasone | 31.9 |
| 16 | Diclofenac | 34.2 |
| 17 | Donepezil | 41.5 |
| 18 | Erythromycin | 38.8 |
| 19 | Etodolac | 30.6 |
| 20 | Fenofibrate | 46.8 |
| 21 | Fluoxetine | 54.9 |
| 22 | Furosemide | 20.1 |
| 23 | Fusidic acid | 36.2 |
| 24 | Gefitinib | 49.1 |
| 25 | Glibenclamide | 32.6 |
| 26 | Glimepiride | 30.8 |
| 27 | Glyburide | 32.6 |
| 28 | Haloperidol | 44.3 |
| 29 | Ibuprofen | 20.6 |
| 30 | Imatinib | 60.9 |
| 31 | Imipramine | 39.4 |
| 32 | Indomethacin | 30.1 |
| 33 | Isoniazid | -10.3 |
| 34 | Itraconazole | 40.6 |
| 35 | Ketoconazole | 41.1 |
| 36 | Leflunomide | 39.0 |
| 37 | Lopinavir | 40.1 |
| 38 | Lovastatin | 44.4 |
| 39 | Miconazole | 53.7 |
| 40 | Nicardipine | 47.0 |
| 41 | Nifedipine | 25.1 |
| 42 | Nitrendipine | 39.4 |
| 43 | Omeprazole | 21.1 |
| 44 | Paclitaxel | 39.2 |
| 45 | Papaverine | 32.0 |
| 46 | Paracetamol | 2.69 |
| 47 | Pinacidil | 29.7 |
| 48 | Pravastatin | 20.5 |
| 49 | Praziquantel | 31.5 |
| 50 | Primaquine | 49.2 |
| 51 | Probenecid | 19.8 |
| 52 | Rifabutin | 43.9 |
| 53 | Rifampicin | 34.7 |
| 54 | Salicylic acid | -8.67 |
| 55 | Saquinavir | 42.2 |
| 56 | Simvastatin | 46.8 |
| 57 | Sulindac | 30.3 |
| 58 | Sulpiride | 7.35 |
| 59 | Telmisartan | 45.6 |
| 60 | Ticlopidine | 48.3 |
| 61 | Trazodone | 32.7 |
| 62 | Verapamil | 44.8 |

**Table S3.** Experimentally determined human serum albumin affinity descriptors expressed as log k_HSA_ values for the 62 compounds included in the dataset. log k_HSA_ was used as a biomimetic chromatographic descriptor of plasma protein binding.

| No. | Compound | log *k*_HSA_ |
| --- | --- | --- |
| 1 | 17-a-Ethinyl-Estradiol | 1.45 |
| 2 | Acitretin | 1.25 |
| 3 | Amiodarone | 1.59 |
| 4 | Amprenavir | 0.78 |
| 5 | Atorvastatin | 1.14 |
| 6 | Benzbromarone | 1.88 |
| 7 | Bezafibrate | 1.19 |
| 8 | Bicalutamide | 1.36 |
| 9 | Buspirone | 0.62 |
| 10 | Caffeine | -0.56 |
| 11 | Chlorpromazine | 1.35 |
| 12 | Clofibrate | 1.27 |
| 13 | Clotrimazole | 1.49 |
| 14 | Dapsone | 0.60 |
| 15 | Dexamethasone | 0.41 |
| 16 | Diclofenac | 1.73 |
| 17 | Donepezil | 0.76 |
| 18 | Erythromycin | -0.22 |
| 19 | Etodolac | 1.25 |
| 20 | Fenofibrate | 1.46 |
| 21 | Fluoxetine | 0.99 |
| 22 | Furosemide | 1.24 |
| 23 | Fusidic acid | 1.32 |
| 24 | Gefitinib | 1.13 |
| 25 | Glibenclamide | 1.52 |
| 26 | Glimepiride | 1.50 |
| 27 | Glyburide | 1.52 |
| 28 | Haloperidol | 0.76 |
| 29 | Ibuprofen | 1.84 |
| 30 | Imatinib | 1.22 |
| 31 | Imipramine | 0.95 |
| 32 | Indomethacin | 1.21 |
| 33 | Isoniazid | -1.48 |
| 34 | Itraconazole | 1.42 |
| 35 | Ketoconazole | 1.18 |
| 36 | Leflunomide | 1.02 |
| 37 | Lopinavir | 1.25 |
| 38 | Lovastatin | 1.24 |
| 39 | Miconazole | 1.54 |
| 40 | Nicardipine | 1.25 |
| 41 | Nifedipine | 0.67 |
| 42 | Nitrendipine | 1.17 |
| 43 | Omeprazole | 1.18 |
| 44 | Paclitaxel | 0.99 |
| 45 | Papaverine | 0.99 |
| 46 | Paracetamol | 0.21 |
| 47 | Pinacidil | 0.14 |
| 48 | Pravastatin | -0.18 |
| 49 | Praziquantel | 0.76 |
| 50 | Primaquine | 0.56 |
| 51 | Probenecid | 1.39 |
| 52 | Rifabutin | 0.46 |
| 53 | Rifampicin | 0.89 |
| 54 | Salicylic acid | 1.01 |
| 55 | Saquinavir | 1.22 |
| 56 | Simvastatin | 1.34 |
| 57 | Sulindac | 1.34 |
| 58 | Sulpiride | -0.35 |
| 59 | Telmisartan | 1.36 |
| 60 | Ticlopidine | 1.45 |
| 61 | Trazodone | 1.09 |
| 62 | Verapamil | 1.00 |

**Table S4.** Calculated physicochemical descriptors for the compounds included in the dataset, obtained using the ACD/Percepta Platform. Descriptors include lipophilicity (log *P*), distribution coefficient at pH 7.4 (log *D*₇.₄), molecular weight (MW), hydrogen-bond donors (HBD), hydrogen-bond acceptors (HBA), topological polar surface area (TPSA), Abraham descriptors (A and B), fractional charge descriptors (fpos: fraction positively charged, fneg: fraction negatively charged, fzw: fraction zwitterionic species) and overall ionization class

| Compound | log *P* | log *D*_7.4_ | MW | HBD | HBA | TPSA, nm^2^ | A | B | fpos | fneg | fzw | Charge |
| --- | --- | --- | --- | --- | --- | --- | --- | --- | --- | --- | --- | --- |
| 17-a-Ethinyl-Estradiol | 3.67 | 3.98 | 296 | 2.00 | 2.00 | 40.5 | 0.90 | 1.02 | 0.00 | 0.00 | 0.00 | Neutral |
| Acitretin | 6.4 | 3.14 | 326 | 1.00 | 3.00 | 46.5 | 0.57 | 0.97 | 0.00 | 1.00 | 0.00 | Acid |
| Amiodarone | 7.80 | 6.38 | 645 | 0.00 | 4.00 | 42.7 | 0.00 | 1.30 | 0.96 | 0.00 | 0.00 | Base |
| Amprenavir | 3.94 | 3.94 | 506 | 4.00 | 9.00 | 140 | 0.64 | 2.61 | 0.00 | 0.00 | 0.00 | Neutral |
| Atorvastatin | 5.19 | 2.22 | 559 | 4.00 | 7.00 | 112 | 1.61 | 2.07 | 0.00 | 1.00 | 0.00 | Acid |
| Benzbromarone | 5.74 | 2.78 | 424 | 1.00 | 3.00 | 50.4 | 0.41 | 0.68 | 0.00 | 1.00 | 0.00 | Acid |
| Bezafibrate | 3.98 | 0.66 | 362 | 2.00 | 5.00 | 75.6 | 0.83 | 1.35 | 0.00 | 1.00 | 0.00 | Acid |
| Bicalutamide | 2.93 | 2.93 | 430 | 2.00 | 6.00 | 116 | 0.71 | 1.63 | 0.00 | 0.00 | 0.00 | Neutral |
| Buspirone | 2.63 | 1.77 | 386 | 0.00 | 7.00 | 69.6 | 0.00 | 2.16 | 0.64 | 0.00 | 0.00 | Weak Base |
| Caffeine | -0.07 | -0.45 | 194 | 0.00 | 6.00 | 58.4 | 0.05 | 1.28 | 0.00 | 0.00 | 0.00 | Neutral |
| Chlorpromazine | 5.35 | 3.34 | 319 | 0.00 | 2.00 | 31.8 | 0.00 | 0.94 | 0.99 | 0.00 | 0.00 | Base |
| Clofibrate | 3.52 | 3.52 | 243 | 0.00 | 3.00 | 35.5 | 0.00 | 0.69 | 0.00 | 0.00 | 0.00 | Neutral |
| Clotrimazole | 4.99 | 5.07 | 345 | 0.00 | 2.00 | 17.8 | 0.00 | 0.83 | 0.58 | 0.00 | 0.00 | Weak Base |
| Dapsone | 0.97 | 1.42 | 248 | 4.00 | 4.00 | 94.6 | 0.45 | 1.35 | 0.00 | 0.00 | 0.00 | Neutral |
| Dexamethasone | 1.83 | 1.80 | 392 | 3.00 | 5.00 | 94.8 | 0.71 | 1.92 | 0.00 | 0.00 | 0.00 | Neutral |
| Diclofenac | 4.40 | 1.23 | 296 | 2.00 | 3.00 | 49.3 | 0.63 | 0.96 | 0.00 | 1.00 | 0.00 | Acid |
| Donepezil | 3.75 | 2.05 | 379 | 0.00 | 4.00 | 38.8 | 0.00 | 1.50 | 0.98 | 0.00 | 0.00 | Base |
| Erythromycin | 2.54 | 0.65 | 734 | 5.00 | 14.00 | 194 | 1.05 | 4.63 | 0.95 | 0.00 | 0.00 | Base |
| Etodolac | 3.81 | -0.05 | 287 | 2.00 | 4.00 | 62.3 | 0.88 | 0.90 | 0.00 | 1.00 | 0.00 | Acid |
| Fenofibrate | 4.38 | 4.38 | 361 | 0.00 | 4.00 | 52.6 | 0.00 | 1.13 | 0.00 | 0.00 | 0.00 | Neutral |
| Fluoxetine | 4.50 | 2.44 | 309 | 1.00 | 2.00 | 21.3 | 0.13 | 0.78 | 1.00 | 0.00 | 0.00 | Base |
| Furosemide | 2.03 | -1.59 | 331 | 4.00 | 7.00 | 131 | 1.35 | 1.45 | 0.00 | 1.00 | 0.00 | Acid |
| Fusidic acid | 5.56 | 2.78 | 517 | 3.00 | 6.00 | 104 | 1.20 | 1.88 | 0.00 | 1.00 | 0.00 | Acid |
| Gefitinib | 4.76 | 4.46 | 447 | 1.00 | 7.00 | 68.7 | 0.25 | 1.87 | 0.53 | 0.00 | 0.00 | Weak Base |
| Glibenclamide | 4.02 | 1.83 | 494 | 3.00 | 8.00 | 122 | 0.85 | 2.01 | 0.00 | 0.99 | 0.00 | Acid |
| Glimepiride | 4.25 | 2.05 | 491 | 3.00 | 9.00 | 133 | 0.75 | 2.15 | 0.00 | 0.99 | 0.00 | Acid |
| Glyburide | 4.02 | 1.83 | 494 | 3.00 | 8.00 | 122 | 0.85 | 2.01 | 0.00 | 0.99 | 0.00 | Acid |
| Haloperidol | 3.36 | 2.18 | 376 | 1.00 | 3.00 | 40.5 | 0.40 | 1.76 | 0.99 | 0.00 | 0.00 | Base |
| Ibuprofen | 3.50 | 0.38 | 206 | 1.00 | 2.00 | 37.3 | 0.59 | 0.81 | 0.00 | 1.00 | 0.00 | Acid |
| Imatinib | 2.22 | 1.81 | 494 | 2.00 | 8.00 | 86.3 | 0.54 | 2.63 | 0.64 | 0.00 | 0.00 | Weak Base |
| Imipramine | 4.28 | 2.61 | 280 | 0.00 | 2.00 | 4.80 | 0.00 | 1.15 | 0.99 | 0.00 | 0.00 | Base |
| Indomethacin | 4.27 | 0.71 | 358 | 1.00 | 5.00 | 68.5 | 0.57 | 1.57 | 0.00 | 1.00 | 0.00 | Acid |
| Isoniazid | -0.81 | -0.81 | 137 | 3.00 | 4.00 | 68.0 | 0.47 | 1.39 | 0.00 | 0.00 | 0.00 | Neutral |
| Itraconazole | 5.66 | 4.32 | 706 | 0.00 | 12.00 | 101 | 0.00 | 2.95 | 0.01 | 0.00 | 0.00 | Neutral |
| Ketoconazole | 4.34 | 3.98 | 531 | 0.00 | 8.00 | 69.1 | 0.00 | 2.22 | 0.15 | 0.00 | 0.00 | Weak Base |
| Leflunomide | 1.59 | 1.59 | 270 | 1.00 | 4.00 | 55.1 | 0.47 | 0.81 | 0.00 | 0.00 | 0.00 | Neutral |
| Lopinavir | 5.16 | 5.16 | 629 | 4.00 | 9.00 | 120 | 0.92 | 2.89 | 0.00 | 0.00 | 0.00 | Neutral |
| Lovastatin | 4.26 | 4.40 | 405 | 1.00 | 5.00 | 72.8 | 0.31 | 1.44 | 0.00 | 0.00 | 0.00 | Neutral |
| Miconazole | 5.34 | 5.45 | 416 | 0.00 | 3.00 | 27.1 | 0.00 | 0.79 | 0.15 | 0.00 | 0.00 | Weak Base |
| Nicardipine | 4.65 | 2.66 | 480 | 1.00 | 9.00 | 117 | 0.13 | 2.12 | 0.41 | 0.00 | 0.00 | Weak Base |
| Nifedipine | 3.27 | 1.12 | 346 | 1.00 | 8.00 | 113 | 0.23 | 1.45 | 1.00 | 0.00 | 0.00 | Base |
| Nitrendipine | 4.15 | 1.6 | 360 | 1.00 | 8.00 | 113 | 0.13 | 1.54 | 0.00 | 0.00 | 0.00 | Neutral |
| Omeprazole | 2.23 | 2.37 | 345 | 1.00 | 6.00 | 96.3 | 0.35 | 2.05 | 0.00 | 0.05 | 0.00 | Neutral |
| Paclitaxel | 3.95 | 3.95 | 854 | 4.00 | 15.00 | 221 | 0.90 | 4.13 | 0.00 | 0.00 | 0.00 | Neutral |
| Papaverine | 2.95 | 3.65 | 339 | 0.00 | 5.00 | 49.8 | 0.00 | 1.47 | 0.07 | 0.00 | 0.00 | Neutral |
| Paracetamol | 0.46 | 0.23 | 151 | 2.00 | 3.00 | 49.3 | 1.04 | 0.86 | 0.00 | 0.00 | 0.00 | Neutral |
| Pinacidil | 2.87 | 2.85 | 245 | 2.00 | 5.00 | 73.1 | 0.39 | 1.54 | 0.04 | 0.00 | 0.00 | Neutral |
| Pravastatin | 2.18 | -1.08 | 425 | 4.00 | 7.00 | 124 | 1.51 | 1.89 | 0.00 | 1.00 | 0.00 | Acid |
| Praziquantel | 3.02 | 3.02 | 312 | 0.00 | 4.00 | 40.6 | 0.00 | 1.46 | 0.00 | 0.00 | 0.00 | Neutral |
| Primaquine | 2.86 | -0.34 | 259 | 3.00 | 4.00 | 60.2 | 0.34 | 1.49 | 1.00 | 0.00 | 0.00 | Base |
| Probenecid | 3.21 | -0.22 | 285 | 1.00 | 5.00 | 83.1 | 0.57 | 1.29 | 0.00 | 1.00 | 0.00 | Acid |
| Rifabutin | 4.50 | 1.86 | 847 | 5.00 | 15.00 | 206 | 1.31 | 4.39 | 0.81 | 0.00 | 0.18 | Weak Base |
| Rifampicin | 2.39 | 1.68 | 823 | 6.00 | 16.00 | 220 | 2.55 | 4.66 | 0.64 | 0.00 | 0.14 | Weak Base |
| Salicylic acid | 2.26 | -1.89 | 138 | 2.00 | 3.00 | 57.5 | 0.71 | 0.38 | 0.00 | 1.00 | 0.00 | Acid |
| Saquinavir | 3.77 | 3.67 | 671 | 6.00 | 11.00 | 167 | 1.46 | 3.89 | 0.22 | 0.00 | 0.00 | Weak Base |
| Simvastatin | 4.87 | 4.87 | 419 | 1.00 | 5.00 | 72.8 | 0.31 | 1.45 | 0.00 | 0.00 | 0.00 | Neutral |
| Sulindac | 3.42 | 0.26 | 356 | 1.00 | 3.00 | 73.6 | 0.57 | 1.39 | 0.00 | 1.00 | 0.00 | Acid |
| Sulpiride | 0.62 | -1.28 | 341 | 3.00 | 7.00 | 110 | 0.72 | 2.15 | 0.98 | 0.01 | 0.00 | Base |
| Telmisartan | 7.44 | 4.06 | 515 | 1.00 | 6.00 | 72.9 | 0.60 | 1.95 | 0.00 | 0.93 | 0.07 | Acid |
| Ticlopidine | 3.68 | 3.38 | 264 | 0.00 | 1.00 | 31.5 | 0.00 | 0.62 | 0.53 | 0.00 | 0.00 | Weak Base |
| Trazodone | 3.80 | 4.60 | 372 | 0.00 | 6.00 | 42.4 | 0.00 | 1.92 | 0.22 | 0.00 | 0.00 | Weak Base |
| Verapamil | 3.83 | 3.17 | 455 | 0.00 | 6.00 | 64.0 | 0.00 | 1.89 | 0.98 | 0.00 | 0.00 | Base |

References

[1] S. Dawson, S. Stahl, N. Paul, J. Barber, J.G. Kenna. *In vitro* inhibition of the bile salt export pump correlates with risk of cholestatic drug-induced liver injury in humans. *Drug Metabolism and Disposition* **40** (2012) 130-138. <https://dx.doi.org/10.1124/dmd.111.040758>
[2] R.E. Morgan, C.J. van Staden, Y. Chen, N. Kalyanaraman, J. Kalanzi, R.T. Dunn, C.A. Afshari, H.K. Hamadeh. A multifactorial approach to hepatobiliary transporter assessment enables improved therapeutic compound development. *Toxicological Sciences* **136** (2013) 216-241. <https://dx.doi.org/10.1093/toxsci/kft176>
[3] J.M. Pedersen, P. Matsson, C.A. Bergström, J. Hoogstraate, A. Norén, E.L. LeCluyse, P. Artursson. Early identification of clinically relevant drug interactions with the human bile salt export pump (BSEP/ABCB11). *Toxicological Sciences* **136** (2013) 328-343. <https://dx.doi.org/10.1093/toxsci/kft197>
[4] F. Montanari, M. Pinto, N. Khunweeraphong, K. Wlcek, M.I. Sohail, T. Noeske, S. Boyer, P. Chiba, B. Stieger, K. Kuchler, G.F. Ecker. Flagging Drugs That Inhibit the Bile Salt Export Pump. *Molecular Pharmaceutics* **13** (2016) 163-171. <https://dx.doi.org/10.1021/acs.molpharmaceut.5b00594>
